# Brugada Phenocopy Associated With Toxic Myocarditis due to Aluminum Phosphide: A Case Report

**DOI:** 10.1155/cric/8858716

**Published:** 2025-08-22

**Authors:** Maria V. Manrique-Marquez, Jorge P. Juarez-Lloclla, Christian A. Rodríguez-Saldaña, Franco León-Jiménez

**Affiliations:** ^1^Department of Internal Medicine, Peru–Korea Santa Rosa II-2 Hospital, Piura, Peru; ^2^Department of Cardiology, Peru–Korea Santa Rosa II-2 Hospital, Piura, Peru; ^3^Department of Internal Medicine, José Cayetano Heredia III-1 Hospital, EsSalud, Piura, Peru

**Keywords:** Brugada phenocopy, cardiac arrhythmias, heart conduction system diseases, myocarditis

## Abstract

We present the case of a 27-year-old woman who ingested aluminum phosphide (AlP) in a suicide attempt, leading to severe toxic myocarditis and a transient Brugada pattern (Brugada phenocopy [BrP]) on her electrocardiogram (ECG). The initial treatment included supportive measures and management of a non-ST elevation acute coronary syndrome. Despite the severity of her condition, the patient stabilized, with normalization of ECG findings and improvement in left ventricular function. This case highlights the importance of recognizing BrP as a potential manifestation of toxic myocarditis, especially in the context of AlP poisoning, and underscores the critical need for early intervention and appropriate management.

## 1. Introduction

Metal phosphides are widely used pesticides that have led to an increase in poisoning cases due to their low cost, wide distribution, and easy access, especially in many countries. Poisoning, whether accidental (occupational) or intentional (suicidal), carries high mortality rates, exceeding 50% [1]. Aluminum phosphide (AlP), a highly toxic rodenticide mainly used for grain storage, causes symptoms almost immediately after ingestion, starting with gastrointestinal distress and progressing to multiorgan failure and refractory shock, leading to death within 24 h to 4 days [1, 2]. Cardiovascular involvement, including toxic myocarditis, refractory heart failure, and rare electrocardiographic changes such as Brugada phenocopy (BrP), is the main cause of death [2, 3].

## 2. Case Report

A 27-year-old woman with no known medical history ingested AlP in a suicide attempt, presenting with vomiting, asthenia, and general malaise. She arrived at the emergency room unconscious, with a blood pressure (BP) of 90/50 mmHg, a heart rate (HR) of 137 bpm, a respiratory rate (RR) of 20 bpm, and an oxygen saturation (SpO_2_) of 94% (FiO_2_ 21%). Lab tests showed the following: WBC 20,220/mm^3^ (Ref: 4000–11,000), hemoglobin 11.6 g/dL (Ref: 12.0–15.0 for females), platelets 208,000/mm^3^ (Ref: 150,000–450,000), creatinine 0.70 mg/dL (Ref: 0.6–1.1 for females), glucose 224 mg/dL (Ref: 70–110), total bilirubin 0.60 mg/dL (Ref: < 1.0), AST 34 U/L (Ref: 10–36), ALT 36 U/L (Ref: 7–35), PT 15.10 s (Ref: 11–13.5), and INR 1.31 (Ref: 0.8–1.1). Arterial blood gas analysis revealed a pH of 7.18, a pCO_2_ of 18.1 mmHg, a lactate of 7.9 mmol/L, and mildly altered electrolytes (Table 1). She received supportive measures including oxygen therapy, hydration, and vasopressor infusion in the intermediate care unit (ICU), leading to clinical improvement.

**Figure 1:**
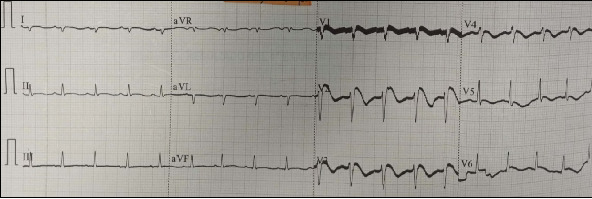
ECG showing Brugada pattern Type 1 on the third day of intoxication, associated with chest pain.

**Figure 2:**
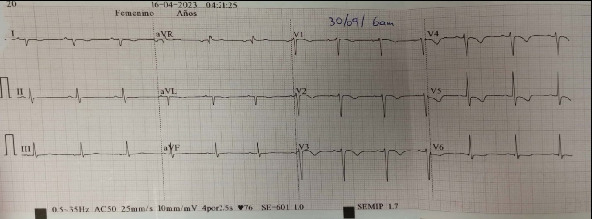
Brugada pattern resolved after 24 h, with persistent negative T waves in the precordial leads.

Three days later, she developed acute and severe chest pain radiating to the left scapular region. Her BP was 85/57 mmHg, HR 110 bpm, RR 19 bpm, and SpO_2_ 96% (FiO_2_ 21%). Cardiac auscultation revealed rhythmic and regular heart sounds, with no significant findings on the rest of the physical exam. Her electrocardiogram (ECG) showed sinus rhythm at 108 bpm, a downsloping ST-segment elevation > 2 mm followed by negative T waves in V1–V3, consistent with a Brugada Type 1 pattern and diffuse repolarization abnormalities (Figure 1). Troponin I was 2.07 ng/mL (Ref: 0.0–0.3), and CK-MB was 245.1 U/L (Ref: < 170 for females). She was diagnosed with a non-ST elevation acute coronary syndrome and treated with antiplatelet therapy, statin, and anticoagulation. Transthoracic echocardiography revealed segmental wall motion abnormalities (anteroseptal and lateral hypokinesia) with a left ventricular ejection fraction (LVEF) of 45%. Coronary computed tomography angiography demonstrated angiographically normal coronary arteries, with no evidence of obstructive lesions and a calcium score of 0. After 24 h, she was asymptomatic, and the ECG showed resolution of the Brugada pattern, with persistent negative T waves in the precordial leads (Figure 2). By Day 9, follow-up echocardiography showed preserved contractility with an LVEF of 59%. She was discharged after psychiatric evaluation, diagnosed with BrP associated with toxic myocarditis from suicidal AlP poisoning.

## 3. Discussion

The extreme toxicity of AlP occurs after exposure to moisture or gastric acid, converting it into phosphine gas, which is primarily absorbed through the lungs [2, 3]. This mitochondrial toxin disrupts cellular function, with hypotheses suggesting a reduction in ATP production and an increase in reactive oxygen species due to inhibition of cytochrome C oxidase Complex IV and other mitochondrial complexes [1]. This leads to cell death and impairment of enzymatic functions in vulnerable organs such as the heart, lungs, liver, and kidneys.

Shadnia and colleagues conducted a 7-year study in Iran, finding that the average age of intoxicated patients was 25.5 years, with intentional oral ingestion being the most common cause (93%) [1]. Clinical manifestations vary, but gastrointestinal symptoms are the most frequent (vomiting in 43% of cases) [1], as seen in our patient and in other cases [2–5]. It has been noted that all patients admitted with a pH < 7.2 die, making it a predictor of mortality [1–3]. However, our patient, with a pH of 7.18, survived, possibly due to a lower dose of phosphide and early supportive treatment. Significant differences between survivors and nonsurvivors include HCO_3_ values (< 11 mEq/L) and ECG abnormalities at admission, although they were not directly correlated with mortality [1]. Our patient had a HCO_3_ of 6.8 mEq/L and ECG abnormalities but survived, possibly due to genetic (ethnic) differences in response to poisoning.

Cardiac damage is more severe due to factors that make the heart particularly susceptible to mitochondrial injury by phosphine: Cardiomyocytes are rich in mitochondria, derive 90% of their energy from mitochondrial processes, and make up 75% of cardiac tissue, with a limited antioxidant system [3, 4]. Cardiovascular manifestations include myocarditis, congestive heart failure, left ventricular (LV) systolic dysfunction, refractory hypotension, and arrhythmias, which are the main causes of death when combined [6]. AlP causes direct myocardial damage, leading to toxic myocarditis, which typically presents with global LV hypokinesia and reduced LVEF on echocardiography, occurring within the first 1–4 days in up to 50% of poisoning cases [5, 6]. Our patient had an LVEF of 45% with segmental wall motion abnormalities on echocardiography, confirming myocarditis. This pattern of wall motion abnormality could represent a novel manifestation of this type of poisoning. Unfortunately, cardiac magnetic resonance imaging was not available at our center, so the diagnosis relied on echocardiographic findings and clinical evolution.

In addition, electrocardiographic changes were observed, including persistent diffuse T-wave inversions associated with myocarditis. Based on this new information, we propose the presence of two phases of clinical manifestations in this type of poisoning: an early phase (< 24 h) and a late phase (≥ 72 h), which may be expected, resembling the three-phase progression described in yellow phosphorus poisoning [7]. This biphasic pattern may be explained by a mechanism of depot effect or slow absorption of AlP in our case, warranting close clinical monitoring.

BrP has recently been defined as cases presenting with a Type 1 or 2 Brugada ECG pattern associated with an identifiable and reversible underlying condition, with normalization of the ECG once the condition resolves [7–9]. In contrast, Brugada syndrome (BrS) is an irreversible genetic disorder classified among channelopathies, which predisposes individuals to ventricular arrhythmias and sudden cardiac death [8]. In our patient, the ECG during the episode of chest pain showed a Type 1 Brugada pattern with diffuse repolarization abnormalities, which later normalized. BrP is most commonly triggered by metabolic disturbances (e.g., hyperkalemia), myocardial ischemia, and pulmonary embolism, while less frequent causes include mechanical compression (e.g., tumors), myocarditis, and pericarditis [9, 10]. Exceptionally rare cases related to AlP poisoning [2–4] should be considered within the broader category of “toxicological causes,” possibly due to direct sodium channel injury in the right ventricular outflow tract. Moreover, our case is the first to report QTc prolongation associated with AlP poisoning, similar to what has been described in yellow phosphorus toxicity, which predisposes to ventricular arrhythmias such as torsades de pointes [11]. We contribute a new case to the literature, extremely rare, consistent with BrP subclassified as Type 1B [9], associated with toxic myocarditis from AlP poisoning.

## 4. Conclusion

AlP is a highly toxic rodenticide frequently used in rural suicide attempts. Its acute toxicity requires prompt recognition and immediate management, as no antidote is currently available. High mortality rates are associated with cardiovascular complications, particularly toxic myocarditis, which can lead to LV dysfunction. Electrocardiographic abnormalities may also occur, including rare transient Brugada patterns.

## Figures and Tables

**Table 1:** Evolution of arterial blood gas and electrolyte parameters during hospitalization in a patient with aluminum phosphide poisoning.

**Parameter (unit) [reference range]**	**Day 0 (hospital admission)**	**Day 3 (BrP ECG)**	**Day 4 (normal ECG)**
PH [7.35–7.45]	7.18	7.28	7.38
Bicarbonate (mmol/L) [22–26]	6.8	12	22
Sodium (mmol/L) [135–145]	138	138	138
Potassium (mmol/L) [3.5–5.0]	3.2	3.5	3.6
Chloride (mmol/L) [98–108]	118	108	113
Calcium (mmol/L) [1.1–1.4]	1.12	1.17	1.13
Lactate (mmol/L) [0.5–2.0]	7.9	4.3	2.2

## Data Availability

The data that support the findings of this case report are available from the corresponding author upon reasonable request. The data are not publicly available due to privacy or ethical restrictions.

## References

[1] Shadnia S., Sasanian G., Allami P. (2009). A Retrospective 7-Years Study of Aluminum Phosphide Poisoning in Tehran: Opportunities for Prevention. *Human & Experimental Toxicology*.

[2] Allam P., Shakya S., Yadav V. (2023). Induction of Brugada Electrocardiogram Pattern With Aluminum Phosphide Poisoning: A Case Report. *Annals of Medicine & Surgery*.

[3] Guru S., Kumar R., Behera A., Patra S., Kumar P. (2020). Aluminium Phosphide-Induced Expression of Covertly Present Brugada Pattern in Electrocardiogram: A Rare Case Report. *Cureus*.

[4] Loddé B., Lucas D., Letort J. M., Jegaden D., Pougnet R., Dewitte J. D. (2015). Acute Phosphine Poisoning on Board a Bulk Carrier: Analysis of Factors Leading to a Fatal Case. *Journal of Occupational Medicine and Toxicology*.

[5] Nayyar S., Nair M. (2009). Brugada Pattern in Toxic Myocarditis Due to Severe Aluminum Phosphide Poisoning. *Pacing and Clinical Electrophysiology*.

[6] Mehrpour O., Asadi S., Yaghoubi M. A., Azdaki N., Mahmoodabadi N., Javadmoosavi S. (2019). Cardiogenic Shock due to Aluminum Phosphide Poisoning Treated With Intra-Aortic Balloon Pump: A Report of Two Cases. *Cardiovascular Toxicology*.

[7] Gottschalk B. H., Anselm D. D., Brugada J. (2016). Expert Cardiologists Cannot Distinguish Between Brugada Phenocopy and Brugada Syndrome Electrocardiogram Patterns. *Europace*.

[8] Baranchuk A., Nguyen T., Ryu M. H. (2012). Brugada Phenocopy: New Terminology and Proposed Classification. *Annals of Noninvasive Electrocardiology*.

[9] Çinier G., Tse G., Baranchuk A. (2020). Brugada Phenocopies: Current Evidence, Diagnostic Algorithms and Future Perspectives. *Turk Kardiyoloji Dernegi Arsivi-Archives of the Turkish Society of Cardiology*.

[10] Adytia G. J., Sutanto H. (2024). Brugada Phenocopy vs. Brugada Syndrome: Delineating the Differences for Optimal Diagnosis and Management. *Current Problems in Cardiology*.

[11] Dharanipradab M., Viswanathan S., Kumar G. R., Krishnamurthy V., Stanley D. D. (2018). Yellow Phosphorus-Induced Brugada Phenocopy. *Journal of Electrocardiology*.

